# Assessing heterogeneous effects and their determinants via estimation of potential outcomes

**DOI:** 10.1007/s10654-019-00551-0

**Published:** 2019-08-16

**Authors:** Anton Nilsson, Carl Bonander, Ulf Strömberg, Jonas Björk

**Affiliations:** 1 EPI@LUND, Lund University, Box 157, 221 00 Lund, Sweden; 2 Centre for Economic Demography, Lund University School of Economics and Management, Box 7083, 220 07 Lund, Sweden; 3 Health Metrics Unit, Sahlgrenska Academy, University of Gothenburg, Box 463, 505 30 Gothenburg, Sweden; 4 Clinical Studies Sweden, Forum South, Skåne University Hospital, 221 85 Lund, Sweden

**Keywords:** Heterogeneity, Potential outcomes, Causal inference, Imputation

## Abstract

When analyzing effect heterogeneity, the researcher commonly opts for stratification or a regression model with interactions. While these methods provide valuable insights, their usefulness can be somewhat limited, since they typically fail to take into account heterogeneity with respect to many dimensions simultaneously, or give rise to models with complex appearances. Based on the potential outcomes framework and through imputation of missing potential outcomes, our study proposes a method for analyzing heterogeneous effects by focusing on treatment effects rather than outcomes. The procedure is easy to implement and generates estimates that take into account heterogeneity with respect to all relevant dimensions at the same time. Results are easily interpreted and can additionally be represented by graphs, showing the overall magnitude and pattern of heterogeneity as well as how this relates to different factors. We illustrate the method both with simulations and by examining heterogeneous effects of obesity on HDL cholesterol in the Malmö Diet and Cancer cardiovascular cohort. Obesity was associated with reduced HDL in almost all individuals, but effects varied with smoking, risky alcohol consumption, higher education, and energy intake, with some indications of non-linear effects. Our approach can be applied by any epidemiologist who wants to assess the role and strength of heterogeneity with respect to a multitude of factors.

## Introduction

When estimating the relationship between an exposure and an outcome, the researcher typically aims at determining an average effect. While such average effects are certainly informative, effects may be vastly different across different subgroups of individuals. Advices and recommendations based only on average responses may be far from optimal from a given individual’s point of view and could even be harmful if the individual response is of the opposite sign. In contrast to traditional approaches, however, there are currently some attempts to shift the focus from average effects to heterogeneous ones, and the growing field of personalized and precision medicine aims to tailor treatments to biomarkers or other characteristics of the individual [1–4].

When researchers try to examine heterogeneous effects of a treatment, an exposure, or a behavior, they usually do so either by stratifying or by introducing interaction terms in a regression model. We argue that these approaches are not ideal for a serious examination of the role of heterogeneity. When stratifying, for example, the researcher would typically split the sample only along one dimension (e.g., the two sexes). But heterogeneity may operate along many dimensions at the same time. Results based on stratification with respect to one dimension will at best provide an incomplete picture and at worst provide faulty conclusions in situations of multiple sources of heterogeneity. Indeed, stratifying with respect to several dimensions is possible but may give rise to very small sample sizes and it can be difficult to make sense of the large number of results.

Introducing interaction terms in a regression model has similar disadvantages. Only interacting with respect to one dimension may be overly simplistic, while interacting with respect to several can give rise to models with complex appearances. Additionally, both stratification and interaction face limitations in that the overall role of heterogeneity is not directly assessable. For example, although tests such as LR can be used to detect whether there is *any* interaction present in a regression model, the overall distribution of treatment effects is not visible.

In this article, we propose a methodology for the assessment of heterogeneity. In addition to providing the researcher with easy-to-interpret estimates of how different factors may influence the effect, the method provides a novel way to illustrate the general heterogeneity in effects graphically. Based on the Rubin Causal Model [5, 6], the method can be interpreted as an imputation of missing values on counterfactual outcomes, followed by a calculation of individual-level effects. Imputation of potential outcomes has been proposed by a few authors before [6–14] but has not yet been widely applied. While some discuss estimation of heterogeneous effects [6, 11, 14], we are not aware of any application in a medical context. Moreover, previous methods are largely based on Bayesian inference. Our method is easy to implement and while it cannot uncover how unobserved factors contribute to heterogeneity, it allows for straightforward examinations of how different observed variables influence the size of the effect.

We use both a simple simulation and a real-world example to illustrate the approach. Results from simulations show that estimates from the model are just as accurate as those from an interaction model. In the real-world example, we examined the heterogeneity in the effects of obesity on HDL levels, finding that obesity was associated with reduced HDL in almost all individuals, but effects varied with smoking, risky alcohol consumption, tertiary education, and energy intake.

## Potential outcomes framework

In the Rubin Causal Model, every individual, *i*, is postulated to have two potential outcomes: $$Y_{0i}$$, which is a theoretical outcome if not treated (*A* = 0); and $$Y_{1i}$$, which is a theoretical outcome if treated (*A* = 1). $$Y_{0i}$$ and $$Y_{1i}$$ may, in turn, may be functions of explanatory variables $$X_{ji}$$. Under the assumption of causal consistency, the actual observation, $$Y_{i}$$, is equal to the potential outcome corresponding to the individual’s treatment status (i.e., $$Y_{i} =$$$$Y_{0i}$$ if $$A_{i} = 0$$ and $$Y_{i} = Y_{1i}$$ if $$A_{i} = 1$$). We here assume that there are no measurement errors in neither the outcome nor in the explanatory variables. Note that “treatment” does not necessarily refer to medical treatment but is interchangeable with “exposure.” The unobserved potential outcome is often referred to as the “counterfactual outcome.”

Only one out of the two potential outcomes can ever be observed, as individuals can only be either treated or untreated. This, in turn, means that the individual treatment effect (ITE), $Y_{1i} - Y_{0i}$ cannot be observed. Estimates may be made, however, and a common goal of statistical methods is to estimate some population parameter corresponding to ITEs, such as the average treatment effect (ATE) [15, 16], also known as the average causal effect, [17–19]. Consistent estimates of such parameters can be obtained under certain assumptions, particularly the “exchangeability” or “ignorability” assumption, postulating that treatment status is independent of potential outcomes, conditionally on variables accounted for in the analysis [6, 20].

We propose an approach, where the two potential outcomes are modelled separately and explicitly, allowing for an examination of their difference. The two potential outcomes can be modelled with a general and flexible model, potentially with complex interactions. Typically, the researcher will not have to report or examine the parameter estimates from this model, and a complex structure is therefore unproblematic, at least to the extent that overfitting is avoided. After obtaining adjusted estimates of treatment effects at the individual level, the researcher then turns to examining these effects, and in particular relate them to sets of explanatory variables, thus uncovering the determinants of heterogeneous effects.

In a linear model, equations for the potential outcomes take the following forms:

1 $$Y_{0i} = \alpha_{0} + \mathop \sum \limits_{j = 1}^{J} \beta_{j0} X_{ji} + \varepsilon_{0i}$$

and

2 $$Y_{1i} = \alpha_{1} + \mathop \sum \limits_{j = 1}^{J} \beta_{j1} X_{ji} + \varepsilon_{1i} .$$

$X_{ji}$ represent all observed variables that are associated with the potential outcomes and the two $\varepsilon$ terms represent unobserved factors. In both equations, we assume no unobserved confounding, which means that the error terms are assumed to have means of zero conditional on covariates $X$. The two error terms can be correlated with each other, although the degree of correlation is unknown as we cannot observe both $Y_{0}$ and $Y_{1}$ for the same individual. In general, the error terms may also have different variances.

For simplicity, we will assume that error terms are not correlated across individuals (no autocorrelation) and that their variances do not depend on *X* (no heteroscedasticity). In principle, these assumptions could be relaxed. Estimation of Eqs. (1) and (2) will entail the first step of our proposed method.

The (individual) treatment effect is given by the difference between Eqs. (2) and (1); that is, by

3 $$ITE = \Delta \alpha + \mathop \sum \limits_{j = 1}^{J} (\Delta \beta_{j} )X_{ji} + \Delta \varepsilon_{i} .$$

Here, the first term $\Delta \alpha$ represents an effect independent of individual characteristics, thus an overall effect of treatment. More specifically, it is the expected treatment effect among those with all covariates being equal to zero, which can be interpreted as the treatment effect for a “typical” individual if continuous covariates are centered and the reference category of any factor variable represents a “typical” person.

The second set of terms $(\Delta \beta_{j} )X_{ji}$ represents effects that can be explained by observable characteristics, and the last term $\Delta \varepsilon_{i}$ represents effects that cannot be explained by observable characteristics. Ideally, although hardly ever realistic, the researcher is able to measure all variables that give rise to heterogeneity. In this scenario, the ITE is fully determined by observable characteristics, meaning that the two error terms are equal so that their difference cancels. We would then get the following expression for the individual treatment effect:

4 $$ITE = \Delta \alpha + \mathop \sum \limits_{j = 1}^{J} (\Delta \beta_{j} )X_{ji} .$$

Since measuring all factors that give rise to heterogeneity is generally unrealistic, the researcher can instead consider *expected* treatment effects, i.e., conditional average treatment effects given covariates. These are found by taking the expectation of Eq. (3), yielding:

5 $$\begin{aligned}E\left[ {ITE|X_{i} } \right] & = E\left[ {\Delta \alpha +\sum\limits_{{j = 1}}^{J} {\left( {\Delta \beta _{j} } \right)}X_{{ji}} + \Delta \varepsilon _{i} |X_{i} } \right] \\ & =\Delta \alpha + \sum\limits_{{j = 1}}^{J} {\left( {\Delta \beta_{j} } \right)} X_{{ji}} ,\end{aligned}$$

where the last equality follows from the assumption that error terms have means of zero conditional on covariates, as in Eqs. (1) and (2). The analysis is then about the component of the overall heterogeneity that can be explained by observable factors. The right-hand sides of Eqs. (4) and (5) are obviously equal and results obtained from our model can in principle be interpreted in terms of either of these, although we generally recommend the latter. If one wanted to simulate the more general Eq. (3), including the error, untestable assumptions on the correlation structure between the two error terms would need to be imposed, and we suspect most researchers may be reluctant to this, although a literature on the topic does exist [9, 14, 21]. Note that the variation in (5) will generally be smaller than that in Eq. (3) as the variance of (3), taken over all covariates *X* as well as the error term can be written $Var[ITE] = Var[\Delta \alpha + \sum\nolimits_{j = 1}^{J} {(\Delta \beta_{j} )} X_{j} + \Delta \varepsilon ] = \sum\nolimits_{j = 1}^{J} {(\Delta \beta_{j} )^{2} } Var[X_{j} ] + Var[\Delta \varepsilon ]$. The variance of Eq. (5) is equal to only the first of the two terms in the final expression, and is thus smaller.

## Estimation procedure

We here describe the method to obtain estimates of (expected) treatment effects and then assess heterogeneous effects. To be specific, our method comprises the following four steps:

1. Estimate regression models for the observed potential outcomes, one for *Y*_0_ and one for *Y*_1_, using a comprehensive set of covariates.
2. Predict both potential outcomes for all individuals by calculating the expected potential outcomes from the corresponding regression, conditional on the covariates in step 1.
3. Calculate the difference between the predicted potential outcomes to obtain the estimated ITE.
4. Regress estimated ITEs on covariates of interest.

The procedure is equivalent to a standard (single) imputation approach, where missing potential outcomes are imputed under the assumption of residuals not varying depending on treatment; see Appendix 1 for a derivation of this result.

## Simulation

We conducted a simple simulation exercise to examine the validity of our approach. We used three covariates, $X_{1}$, $X_{2}$, and $X_{3}$, drawn from a multivariate normal distribution, with means 0, variances 1, and correlations 0.5. Treatment was simulated through a probit model; more specifically, by constructing the sum of $X_{1}$, $X_{2}$, $X_{3}$, and another standard normal variable, and letting *A* = 1 if and only if this sum was less than its 30th percentile. We generated potential outcomes according to Eqs. (1) and (2), with $Y_{0i} = 1 + 2X_{1i} + 3X_{2i} + 4X_{3i} + \varepsilon_{0i}$ and $Y_{1i} = 4 + 3X_{1i} + 3.5X_{2i} + 4X_{3i} + \varepsilon_{1i}$, and error terms coming from standard normal distributions. This implied expected ITEs of

6 $$E\left[ {ITE|X_{i} } \right] = 3 + X_{1i} + 0.5X_{2i} ,$$

i.e., there is no heterogeneity with respect to the third covariate. We simulated 1000 datasets with 500 observations in each, and for statistical inference we bootstrapped the entire procedure using 1000 bootstrap replications per simulation. Predictions of potential outcomes (steps 1–2) were based on linear regression models that simply used the covariates $X_{1}$, $X_{2}$, and $X_{3}$. These were also the covariates we related the estimated treatment effects to (step 4), although in practice the researcher may use a smaller set of covariates in this step compared to when predicting potential outcomes. In Table 1 below, results from our model are contrasted to those from linear regressions with interaction terms, estimated by ordinary least squares (OLS).

**Table 1:** Simulation results: results from the imputation/prediction model in the first column and results from a standard regression model with interactions in the second column

Model	Imputation	Interaction
Average $\widehat{\Delta \alpha }$	3.005	3.005
Average $\widehat{\Delta \beta }_{1}$	1.002	1.002
Average $\widehat{\Delta \beta }_{2}$	0.503	0.503
Average $\widehat{\Delta \beta }_{3}$	− 0.001	− 0.001
Average standard error of $\widehat{\Delta \alpha }$	0.179	0.178
Average standard error of $\widehat{\Delta \beta }_{1}$	0.130	0.130
Average standard error of $\widehat{\Delta \beta }_{2}$	0.130	0.131
Average standard error of $\widehat{\Delta \beta }_{3}$	0.130	0.130
95% CI coverage $\widehat{\Delta \alpha }$	0.955	0.955
95% CI coverage $\widehat{\Delta \beta }_{1}$	0.949	0.952
95% CI coverage $\widehat{\Delta \beta }_{2}$	0.951	0.961
95% CI coverage $\widehat{\Delta \beta }_{3}$	0.949	0.954

In the imputation/prediction model, bootstrap was used for statistical inference

Since our model is, in effect, a stratified model (stratification with respect to treatment status), it yielded the very same point estimates as the interaction model. Furthermore, standard errors were very similar and 95% CIs had the supposed coverage not only for the interaction but also for the imputation model. Bootstrap CIs were formed using normal approximation, but we have verified that bootstrap confidence intervals based on percentiles give accurate coverages as well. While it is comforting that our model yields accurate results just like an interaction model, the intuitive interpretation of our model is somewhat different from that of an interaction model, as focus lies on treatment effects rather than the original outcomes. Moreover, since ITEs immediately follow from the model, these can be examined along different dimensions, as we will now illustrate.

**Fig. 1:**
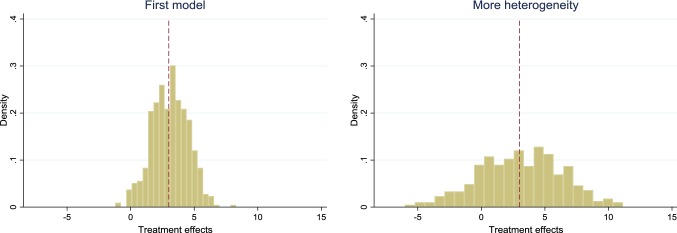
Estimated treatment effects according to simulations in the first model as well as in a model with increased heterogeneity, obtained according to steps 1–4. The average effect is indicated by a dashed line

Based on one particular simulation conducted above, we drew the histogram of estimated treatment effects shown to the left in Fig. 1. Indeed, the figure has roughly the appearance of a normal distribution, which will not necessarily be the case when covariates have other distributions. Also, the appearance will depend on how much variation is induced by differences in model parameters across Eqs. (1) and (2). To the right in Fig. 1, we show an alternative scenario where $Y_{0}$ was generated according to the same scenario as before but $Y_{1i} = 4 + 4X_{1i} + 5X_{2i} + 4X_{3i} + \varepsilon_{1i}$, which means that more heterogeneity was introduced, as the differences in the impacts of covariates on $Y_{1}$ and $Y_{0}$ became larger. Indeed, the variation in treatment effects was now larger and the effect varied between − 6 and 11 rather than between − 1 and 8.

**Fig. 2:**
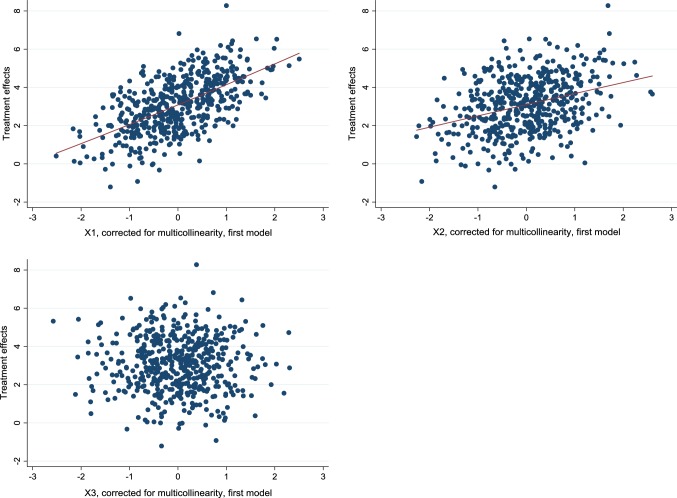
Estimated treatment effects at different levels of covariates according to the first simulation. In the two first curves, fitted quadratic curves have been inserted

In addition to the histogram, it is useful to graphically examine the relationship between treatment effects and covariates in order to (1) get a sense of whether any heterogeneity is present and if so, (2) detect any non-linear effects. For the latter, it is important that nonlinearities are captured by our estimated treatment effects, and we therefore add all quadratic terms and two-way interactions in step 1 of the analysis. Plotting the relationship between estimated treatment effects and $X_{1}$ in our first example, we found the pattern in the first graph Fig. 2, which indeed shows evidence of heterogeneity as the effect varies by the covariate. There is no evidence of non-linearity, as a fitted quadratic curve looks essentially linear. Indeed, the slope of the curve, roughly equal to 1, reflects the estimate of the coefficient of $X_{1}$, $\Delta \beta_{1}$, whereas the average treatment effect of 3 when $X_{1}$ is zero reflects $\Delta \alpha$ in Eq. (6). Correspondingly, as shown in the second graph, the treatment effect increases by 0.5 per increase in $X_{2}$, with no evidence on non-linear effects. Finally, as shown in the third graph, there was no evidence of any heterogeneity with respect to $X_{3}$. Note that, for the figures, we corrected covariates for multicollinearity by regressing them on other covariates and then used the residual from that regression instead of the covariate in question; see Appendix 2 for a derivation of this method.

To examine the robustness of our simulations with respect to imposed assumptions, two additional simulations were carried out (see Appendix 3). In the first of these (Table 4) we increased the correlation between the first two covariates from 0.5 to 0.9. Again, results are virtually identical to those from a linear regression model with interactions.

In the second additional simulation (Table 5), we again increased the correlation between the first two covariates to 0.9 but additionally omitted the second covariate from the last step of the model. This is in line with our suggestion that a more comprehensive set of variables may be used for the estimation of potential outcomes, and a smaller set of “relevant” covariates in the final estimation step. In the case of our simulation, we can imagine that the two first covariates measure roughly the same entity of interest, and one is dropped to avoid multicollinearity. As can be seen, parameter estimates from this simulation are biased, as compared to the “correct” parameters in Eq. (6). The biases in our model differ somewhat from those generated by an interaction model, while standard errors are very similar across the models (with the standard error of $\widehat{\Delta \beta }_{1}$ now being much lower than in the previous simulation).

The different biases in the estimated effect of $X_{1}$ when omitting $X_{2}$ either from our model or from the interaction model can be determined using the “omitted variables formula” [22]. According to this formula, the bias that occurs when dropping a variable from a linear regression is determined by the product of two factors: (1) the effect of the omitted variable on the outcome and (2) the “effect” of the included variable of interest on the excluded one (controlling for all included variables). With our model, the bias in the effect of $X_{1}$ on the ITE is thus given by the product of 0.5 (which is the effect of $X_{2}$ on ITE) and the “effect” of $X_{1}$ on $X_{2}$, controlling for $X_{3}$. Correspondingly but somewhat differently, in the interaction model, the bias in the $A*X_{1}$ on $Y$ is given by the product of 0.5 (which is the effect of $A*X_{2}$ on $Y$) and the “effect” of $A*X_{1}$ on $A*X_{2}$, controlling for all main effects and the interaction $A*X_{3}$. The second factor of the omitted variables formula thus differs and has a simpler determination with our approach.

## A real-world example: data and setup

To illustrate our proposed method with a real-world example, we used data from the Malmö Diet and Cancer (MDC) Study [23] to examine heterogeneous effects of baseline obesity (the exposure) on subsequent high-density lipoprotein (HDL), “the good cholesterol” (the outcome). MDC is a prospective population-based cohort, where about 17,000 women (born 1923–1950) and 11,000 men (born 1923–1945) were initially recruited in Malmö, Sweden, between 1991 and 1996. Data represent a combination of self-reports on life-style such as food intake, socioeconomic factors, medications, and previous diseases; as well as measurements of anthropometry and many other factors. In addition, comprehensive register data has been linked from government authorities including Statistics Sweden and the National Board of Health and Welfare.

A subsample of the individuals in MDC formed a cardiovascular cohort and underwent additional examinations and assessments of cardiovascular risk factors. A follow-up was conducted in 2007–2012 and we used data on HDL from this source.

The following variables, based on self-reports, were used in the first step: physical activity energy expenditure (a score based on time spent in different activities per week, similar to the Minnesota Leisure Time Physical Activity Instrument [24]), smoking (yes or no), alcohol intake, fat intake, carbohydrate intake, protein intake, cholesterol intake, and total energy intake. Energy intake was measured in kcal per day whereas other intake variables were measured in grams. We additionally exploited information on age, sex and educational attainment (primary, secondary, or tertiary education). For simplicity, individuals with missing data on relevant variables were discarded (although in a more general setup, such data could be imputed as well).

For the analysis of heterogeneity, presentation of estimates becomes a major point of interest and it is therefore important to use variables that are natural to interpret as well as avoiding variables that capture similar processes, so that multicollinearity arises. We thus created an indicator for “risky alcohol consumption” (more than 60 g/day if male and more than 40 g/day if female). We also created indicators for 10-year age spans. Moreover, we used total energy intake per day and not the more specific variables of protein, fat, carbohydrate, and cholesterol intake (which are all strongly correlated with total energy intake). Continuous independent variables (physical activity and energy intake) were centered so that the intercept may be interpreted as the effect on an individual with average values on these variables and zeros on binary/categorical variables.

The sample contained 3385 individuals with non-missing information on relevant variables (11% were obese, where obesity was defined as having a body mass index (BMI) of 30 or more). Descriptive statistics for variables used in the heterogeneity analysis are shown in Table 2.

**Table 2:** Descriptive statistics for obese and non-obese individuals in the sample

Variable	Non-obese (n = 3028)	Obese (n = 357)
HDL (ln mmol/l)	0.33 (0.30)	0.19 (0.28)
Risky alcohol consumption	1%	2%
Smoker	24%	15%
Physical activity score	8288 (5779)	7170 (5124)
Energy intake (kcal/day)	2342 (657)	2303 (673)
Primary education	68%	76%
Secondary education	11%	5%
Tertiary education	22%	18%
Male	40%	38%
Age 45–49	17%	13%
Age 50–59	52%	50%
Age 60–68	31%	37%

Continuous variables are presented with means and standard deviations whereas binary variables are presented with percentages

## A real-world example: results

**Fig. 3:**
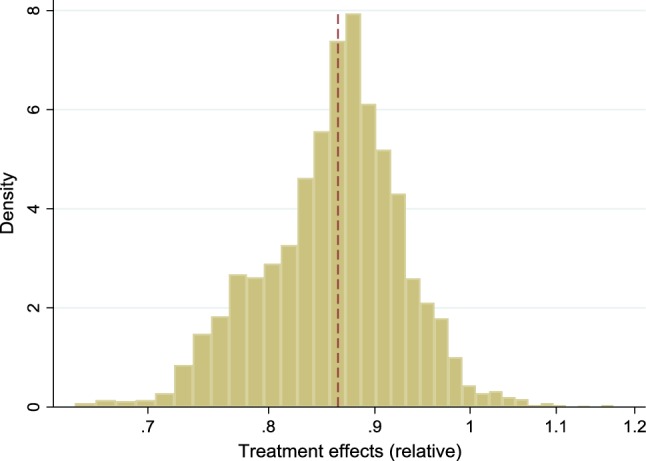
Estimated (relative) effects of obesity on HDL levels in the empirical example, obtained according to steps 1–3. The average effect is indicated by a dashed line

We proceeded as described in steps 1–3 above. Using the logarithm of HDL as the outcome, our estimated ITEs had a mean of − 0.147 (with a median of − 0.139 and an interquartile range of 0.091). Relative effects are obtained by taking the exponential and we find that the relative mean ITE is equal to 0.864. A histogram of relative ITEs is shown in Fig. 3. More than 98% of the effects are less than one, where one represents no effect.

We then regressed treatment effects on covariates (step 4), bootstrapping the entire procedure 1000 times to obtain standard errors. Specifically, we here regressed individuals’ predicted difference in log HDL under obesity and non-obesity on a set of seven variables. This yielded the results in Table 3.

**Table 3:** Relative impacts of individual characteristics on the estimated effects of having BMI ≥ 30 versus BMI < 30 on HDL (ln mmol/l), according to the proposed four-step method

	Univariate (95% CI)	Multivariate (95% CI)
Risky alcohol consumption	1.142 (0.986–1.324)	1.155 (1.000–1.334)
Smoker	0.886 (0.806–0.975)	0.890 (0.807–0.982)
Physical activity score (10,000 units, centered)	1.015 (0.955–1.079)	1.014 (0.954–1.078)
Energy intake (1000 kcal/day, centered)	0.967 (0.926–1.010)	0.949 (0.906–0.995)
Primary education	1.000 (ref)	1.000 (ref)
Secondary education	1.008 (0.906–1.122)	1.018 (0.914–1.134)
Tertiary education	1.081 (1.004–1.163)	1.077 (1.002–1.159)
Male	1.019 (0.961–1.080)	1.051 (0.987–1.120)
Age 45–49	0.995 (0.949–1.043)	1.001 (0.967–1.036)
Age 50–59	1.000 (ref)	1.000 (ref)
Age 60–68	1.001 (0.953–1.052)	0.991 (0.949–1.035)
Constant	–	0.855 (0.817–0.895)

Heterogeneous effects of obesity on HDL levels (ln mmol/l). Normality-based confidence intervals were formed by bootstrapping the entire procedure. Since univariate models (i.e., models with only one variable included in step 4) were estimated separately, there is no common intercept to be reported. Effects are multiplicative, as the outcome is measured in logarithms and we have exponentiated coefficient estimates.

The “constant” of 0.855 shows that in the reference group, obese individuals have a 14.5% lower HDL level than non-obese. This is similar to the average treatment effect of 0.864 previously reported. One should note, however, that individuals in the reference group may deviate from average individuals and in general, the effect on them does not have to be the same as the average effect.

Turning to the heterogeneity analysis (the remaining estimates in Table 3), results suggest that the reducing effects of obesity on HDL may be stronger among individuals that smoke or have higher energy intake, while being weaker among those with university education or with risky alcohol consumption. Effects are multiplicative so, for example, while smoking multiplies the (expected) treatment effect by 0.890 and an increased energy intake by 1000 kcal/day multiplies it by 0.949 according to the multivariate model, the combination of smoking and a 1000 kcal/day higher energy intake multiplies it by 0.890 * 0.949. This yields an (expected) treatment effect of 0.855 * 0.890 * 0.949 = 0.722 for an individual who deviates from the reference group both in terms of being smoker and having a 1000 kcal/day higher energy intake than average.

For comparison, we have estimated the relationship between HDL and obesity with an interaction model, using all the variables that we entered in step 1–3 above as main effects, and the variables that we entered in step 4 interacted with obesity. Estimates corresponding to those in Table 3 (i.e., the interactions and the main effect of obesity but no other main effects) are reported in Table 6 in Appendix 4. As can be seen, results are similar, both in terms of point estimates and confidence intervals.

**Fig. 4:**
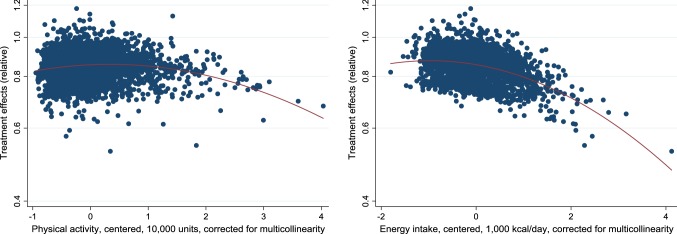
Estimated (relative) effects of obesity on HDL levels by different levels of physical activity or energy intake. Fitted quadratic curves have been inserted (vertically shifted in order for treatment effects at *x* = 0 to represent individuals in the reference group)

We then examined graphically the relationships between ITEs and the continuous covariates: physical activity and energy intake. To do this, we added the squares of physical activity and energy intake into the first step of the model, estimated the ITEs and then constructed plots of these estimated effects against (centered) physical activity and energy intake. The results are shown in Fig. 4. As before, covariates have been adjusted for multicollinearity by using residuals from regressions of the covariate versus other covariates. In the graphs, we also inserted fitted quadratic curves, in order to highlight the possibility of relationships varying depending on whether the covariate is small or large. While the main purpose of the graphs is to detect variations in slopes, the levels of the curves are of less interest, but we have adjusted them vertically by − 0.015 to make their value at *x* = 0 correspond to the treatment effect of 0.855 for a person in the reference group (see Table 3).

In line with our estimates in Table 3, Fig. 4 suggests no clear relationship between physical activity and treatment effects—the pattern is quite flat. However, the stronger effect of obesity on HDL among those with higher energy intake is clearly confirmed in the second graph, where a downward slope can be seen. A visual inspection of the graphs suggests no clear evidence of non-linearity, although the fitted quadratic curves indicate potentially accelerated effects among individuals with high physical activity or high energy intake. With results like these, the researcher may opt for a more sophisticated model and for example include quadratic terms when estimating determinants of the treatment effects in step 4, although we abstain from this due to power concerns.

## Discussion

Standard statistical models, such as regressions, are typically based on the implicit assumption that effects are homogeneous across individuals. This may often be unrealistic, as individual outcomes are shaped through a complex combination of genetics, environmental exposures, and behaviors. When researchers ignore the possibility of heterogeneous effects they generate evidence that can be misleading for large subgroups of individuals. In this article, we proposed a way of assessing the role of heterogeneity in effects. The method is simple to implement, and results have a straightforward interpretation, as we only estimate main effects (on treatment effects) rather than interactions (as would have been the case in an interaction model). The main prerequisite to apply our method is a basic understanding of the potential outcomes framework, a framework that is gaining increased popularity as attention in epidemiology is shifting towards the estimation of causal effects.

Some readers will notice that our approach has similarities with multilevel/hierarchical models [25, 26], where treatment effects can be modelled as functions of covariates. Such models, however, make the most sense when observations can naturally be divided into groups, a scenario that is generally not at hand, at least not when covariates are continuous. There are also more complex approaches to analyze heterogeneity, such as models based on Bayesian inference, decision trees, and machine learning [27–30]. It is fully possible to implement versions of our approach where, for example, a more advanced strategy is used to obtain ITEs in steps 1–3.

The results from our simulation showed that the model works just as well as a correctly specified interaction model, both in terms of accuracy and precision. The results from our empirical example suggested that the effect of obesity on HDL may be larger in individuals smoke or have a higher energy intake, while being smaller in individuals with higher education or risky alcohol consumption. Graphical illustrations provided easily interpreted summaries of the degree of (explained) variation in effects, and showed some evidence of nonlinearities in the interplay between covariates and obesity.

The possibility to graphically inspect the heterogeneity in treatment is an important feature of our approach. Several different versions of the histograms can be considered, such as with restrictions on covariates or treatment status. Correlation plots of treatment effects versus covariates are also useful for model-building and decisions about functional forms in the last step of the analysis.

As shown in Appendix 1, our approach is equivalent to a (single) imputation approach, where missing potential outcomes are replaced by predicted ones. There is a large and expanding literature on how missing data may be imputed [31–35]. Methods include both single and multiple imputations, where the latter involve replacing each missing value with several imputed. Multiple imputation is often motivated for reasons of statistical inference, although single imputation combined with bootstrap has been shown to perform very well and sometimes better than multiple imputation in standard analyses [32].

It would certainly be possible to carry out our method with multiple imputation as well, imputing unobserved outcomes in Eqs. (1) and (2) and then calculating treatment effects. This would allow for the scenario in Eq. (3), where the two error terms do not cancel. Under such a scenario, the imputed values could either be generated independently (under the extreme assumption of no correlation between the two error terms) or under some other assumption on the correlation structure [14, 21]. However, the structure of the relationship between the error terms cannot be known, as the two terms are never both observed for the same individual. Instead of imposing something arbitrary we therefore believe it to be a useful working assumption to postulate they are equal—or, equivalently to let the entity of interest be expected rather than actual treatment effects. The only consequence of this choice is that the variability in the estimated treatment effects reduces, as the variance of Eq. (4) equals the variance of Eq. (3) minus the variance of $\Delta \varepsilon$. Allowing the error terms to vary within individuals, as in Eq. (3), would increase the variability in the histograms and decrease the precision of estimates, but leave the expected point estimates unchanged. More precisely, the histograms of treatment effects would be more dispersed but one would still obtain consistent parameter estimates under the assumption that error terms have expectations of zero conditional on included covariates, since in Eq. (3), the expected conditional error term can be written $E\left[ {\Delta \varepsilon \left| {X\left] \,= E \right[\varepsilon_{1} - \varepsilon_{0} } \right|X\left] \,= E \right[\varepsilon_{1} \left| {X\left] { - E} \right[\varepsilon_{0} } \right|X} \right] = 0 - 0 = 0.$

Our empirical analysis was explorative and we ignored issues such as mass significance and multiple hypothesis testing. Just like standard analyses of main effects, analyses of heterogeneous effects may want apply multiple hypothesis testing [36–38] and we believe that more research needs to be done on multiple hypothesis testing in the context of heterogeneity. Given our focus on exploration and the illustration of a method, we also abstained from discussions of biological mechanisms.

Our method has some limitations. As in any observational analysis, unobserved confounding cannot be ruled out, and in our context this can mean either confounding of the overall treatment effect or confounded heterogeneity. The causal interpretation of the results relies on the ability to measure the confounding variables—to avoid confounded heterogeneity, particularly any factors that are both related to observed covariates and to the ITE. However, our approach can still be applied to study heterogeneity in association estimates, which is useful for predictions.

As is generally the case, results may also depend on functional form and, in particular, the size and pattern of heterogeneous effects may depend on the scale chosen, e.g., additive or multiplicative scale [39]. In the present study, we have also restricted ourselves to linear models with continuous outcomes and binary treatments, although outcomes in medicine are often binary or time-to-event, and treatments or exposures may not be binary. In future work we aim to address how the approach may be generalized and applied to different types of models with different outcomes.

## Appendix 1

We here show the equivalence of our method (steps 1–3) and a single imputation approach, where the errors from the regressions predicting the two potential outcomes are assumed to be equal. As noted, equality of the errors is reasonable in a well-specified linear model, where all heterogeneity arises from observed factors. We also assume that regressions are estimated on large enough data so that residuals equal errors, implying that the two residuals are equal to each other as well. This assumption may be relaxed if the focus of interest is on expected effects rather than actual ones, since the expected differences between the two residuals would still be zero.

Say that $Y_{0i}$ is observed and that $Y_{1i}$ needs to be imputed. This can be done according to:

7 $$Y_{1i} = \hat{\alpha }_{1} + \mathop \sum \limits_{j = 1}^{J} \hat{\beta }_{j1} X_{ji} + \hat{\varepsilon }_{1i} = \hat{\alpha }_{1} + \mathop \sum \limits_{j = 1}^{J} \hat{\beta }_{j1} X_{ji} + \hat{\varepsilon }_{0i} = \hat{\alpha }_{1} + \mathop \sum \limits_{j = 1}^{J} \hat{\beta }_{j1} X_{ji} + \left( {Y_{0i} - \hat{\alpha }_{0} - \mathop \sum \limits_{j = 1}^{J} \hat{\beta }_{j1} X_{ji} } \right)$$

Taking the difference between $Y_{1i}$ and $Y_{0i}$ then yields

8 $$\begin{aligned}Y_{{1i}} - Y_{{0i}} & = \hat{\alpha }_{1} + \sum\limits_{{j =1}}^{J} {\hat{\beta }_{{j1}} } X_{{ji}} - \hat{\alpha }_{0} -\sum\limits_{{j = 1}}^{J} {\hat{\beta }_{{j0}} } X_{{ji}} \\ &= \Delta \hat{\alpha } + \sum\limits_{{j = 1}}^{J} \Delta\hat{\beta }_{{j1}} X_{{ji}}\end{aligned}$$

This is exactly the expression implied by our method. The same result is also obtained if $Y_{0i}$ is missing whereas $Y_{1i}$ is observed, which can be verified analogously.

## Appendix 2

We here show mathematically why scatterplots with outcomes on the y-axis and residuals from regressions of one covariate on other covariates on the x-axis can be used to detect non-linear effects that do not suffer from confounding. Our approach corresponds to a method that has previously been implemented in STATA [40] and builds on the “regression anatomy formula” [41], according to which a slope coefficient in a multiple linear regression model can just as well be obtained from a simple linear regression of the outcome on the above-mentioned residual.

For concreteness, assume that an outcome is determined by a quadratic polynomial plus an error term, and we want to assess non-linearity with respect to the first covariate $X_{1}$ on the outcome (since the approach is general, we abstain from referring to the outcome as a treatment effect, although this is the outcome modeled in the article). We assume there are *J* main effects and that all two-way interactions are included:

9 $$Y_{i} = a + b_{1} X_{1i} + c_{1} X_{1i}^{2} + \mathop \sum \limits_{j = 2}^{J} d_{1j} X_{1i} X_{ji} + \mathop \sum \limits_{k = 2}^{J} b_{k} X_{ki} + \mathop \sum \limits_{k = 2}^{J} c_{k} X_{ki}^{2} + \mathop \sum \limits_{k = 2}^{J} \mathop \sum \limits_{j > k}^{J} d_{jk} X_{ji} X_{ki} + e_{i} .$$

To ease exposition, the first covariate $X_{1}$, its squared term, and its interactions with other covariates have been broken out of the terms summing over *k*. If $X_{1}$ were independent of all other covariates, plotting the relationship between $Y$ and $X_{1}$ would have been unproblematic; the contributions of covariates other than $X_{1}$ would then tend to be the same regardless of $X_{1}$, thus only adding random noise. Fitting a quadratic curve with no adjustment for confounding, one would obtain the following slope, which is obtained as the derivative of the outcome with respect to the first covariate, averaged over other covariates and the error:

10 $$\frac{{\overline{dY}_{i} }}{{dX_{1i} }} = b_{1} + 2c_{1} X_{1i} + \mathop \sum \limits_{j = 2}^{J} d_{1j} \overline{X}_{ji} .$$

However, with confounding factors, the pattern could look completely different, not reflecting true impacts of $X_{1}$. To proceed, assume that $X_{1}$ is related to other covariates through the linear equation:

11 $$X_{1i} = g + \mathop \sum \limits_{k = 2}^{J} h_{k} X_{ki} + u_{i} .$$

The square of Eq. (11) is:

12 $$X_{1i}^{2} = \left( {g + \mathop \sum \limits_{k = 2}^{J} h_{k} X_{ki} + u_{i} } \right)^{2} = g^{2} + 2g\mathop \sum \limits_{k = 2}^{J} h_{k} X_{ki} + 2gu_{i} + \mathop \sum \limits_{k = 2}^{J} \mathop \sum \limits_{j = 2}^{J} h_{j} h_{k} X_{ji} X_{ki} + 2\left( {\mathop \sum \limits_{k = 2}^{J} h_{k} X_{ki} } \right)u_{i} + u_{i}^{2} .$$

Plugging (11) and (12) into (9) yields:

13 $$\begin{aligned} Y_{i} & = a + b_{1} \left( {g + \mathop \sum \limits_{k = 2}^{J} h_{k} X_{ki} + u_{i} } \right) + c_{1} (g^{2} + 2g\mathop \sum \limits_{k = 2}^{J} h_{k} X_{ki} + 2gu_{i} + \mathop \sum \limits_{k = 2}^{J} \mathop \sum \limits_{j = 2}^{J} h_{j} h_{k} X_{ji} X_{ki} \\ & \quad + 2\left( {\mathop \sum \limits_{k = 2}^{J} h_{k} X_{ki} } \right)u_{i} + u_{i}^{2} ) + \mathop \sum \limits_{j = 2}^{J} d_{1j} \left( {g + \mathop \sum \limits_{k = 2}^{J} h_{k} X_{ji} + u_{i} } \right)X_{ji} + \mathop \sum \limits_{k = 2}^{J} b_{k} X_{ki} \\ & \quad + \mathop \sum \limits_{k = 2}^{J} c_{k} X_{ki}^{2} + \mathop \sum \limits_{k = 2}^{J} \mathop \sum \limits_{j > k}^{J} d_{jk} X_{ji} X_{ki} + e_{i} . \\ \end{aligned}$$

Equation (13) can be viewed as a function of the error $u$, where the latter may be estimated from Eq. (11). Notably, the error is unrelated to all covariates other than $X_{1}$ and as a result, a plot of the outcome $Y$ versus the error will not be distorted by confounding. The derivative of Eq. (13) with respect to the error is:

14 $$\frac{{dY_{i} }}{{du_{i} }} = b_{1} + 2c_{1} \left( {g + \mathop \sum \limits_{k = 2}^{J} h_{k} X_{ki} + u_{i} } \right) + \mathop \sum \limits_{j = 2}^{J} d_{1j} X_{ji} .$$

In the special case where all covariates have means of zero (which implies that $g = 0$), this derivative equals $b_{1} + 2c_{1} \sum\nolimits_{j = 2}^{J} {h_{j} } X_{ji} + 2c_{1} u_{i} + \sum\nolimits_{j = 2}^{J} {d_{1j} } X_{ji} ,$ which after taking averages over all covariates other than the first becomes:

15 $$\frac{{\overline{dY}_{i} }}{{du_{i} }} = b_{1} + 2c_{1} u_{i} .$$

This expression mirrors Eq. (10) in the case where all covariates have means of zero, implying that Eq. (15) can be used for an analysis of the effects of $X_{1}$ on the outcome.

If $X_{1}$ has mean zero whereas other covariates may not, then $g = - \sum\nolimits_{k = 2}^{K} {h_{k} } \bar{X}_{ki}$ and Eq. (14) becomes:

16 $$\begin{aligned} \frac{{dY_{i} }}{{du_{i} }} & = b_{1} + 2c_{1} \left( { - \sum\limits_{{k = 2}}^{J} {h_{k} } \bar{X}_{{ki}} + \sum\limits_{{k = 2}}^{J} {h_{k} } X_{{ki}} + u_{i} } \right) \\ & \quad + \,\sum\limits_{{j = 2}}^{J} {d_{{1j}} } X_{{ji}}. \end{aligned}$$

After averaging over all covariates other than the first, this equation mirrors (10), as desired.

In the case where also $X_{1}$ may not be non-zero on average, a plot of the outcome versus the residuals will still give the accurate slopes, given that we consider the plot as horizontally shifted. To see this, suppose we generate another variable $\widetilde{u}_{i} = u_{i} + \overline{X}_{1i} = u_{i} + g + \mathop \sum \nolimits_{j = 2}^{J} h_{j} \overline{X}_{ji}$, which is simply the error term shifted to the right by the average of $X_{1}$. The error $u$ can now be written $u_{i} = \tilde{u}_{i} - g - \mathop \sum \nolimits_{j = 2}^{J} h_{j} \overline{X}_{ji}$, and applying the chain rule, the derivative of the outcome with respect to $\widetilde{u}$ is determined as:

17 $$\begin{aligned} \frac{{dY_{i} }}{{d\tilde{u}_{i} }} & = \frac{{dY_{i} }}{{du_{i} }}\frac{{du_{i} }}{{d\tilde{u}_{i} }} = \left( {b_{1} + 2c_{1} \left( {g + \mathop \sum \limits_{k = 2}^{J} h_{k} X_{ki} + u_{i} } \right) + \mathop \sum \limits_{j = 2}^{J} d_{1j} X_{ji} } \right)*1 \\ & = b_{1} + 2c_{1} \left( {g + \mathop \sum \limits_{k = 2}^{J} h_{k} X_{ki} + u_{i} } \right) + \mathop \sum \limits_{j = 2}^{J} d_{1j} X_{ji} \\ & = b_{1} + 2c_{1} \left( {g + \mathop \sum \limits_{k = 2}^{J} h_{k} X_{ki} + \left( {\tilde{u}_{i} - g - \mathop \sum \limits_{j = 2}^{J} h_{j} \overline{X}_{ji} } \right)} \right) + \mathop \sum \limits_{j = 2}^{J} d_{1j} X_{ji} \\ & = b_{1} + 2c_{1} \left( {\mathop \sum \limits_{k = 2}^{J} h_{k} X_{ki} + \tilde{u}_{i} - \mathop \sum \limits_{k = 2}^{J} h_{k} \overline{X}_{ki} } \right) + \mathop \sum \limits_{j = 2}^{J} d_{1j} X_{ji} . \\ \end{aligned}$$

On average (averaging over all covariates except the first) this expression becomes:

18 $$\frac{{\overline{dY}_{i} }}{{d\tilde{u}_{i} }} = b_{1} + 2c_{1} \tilde{u}_{i} + \mathop \sum \limits_{j = 2}^{J} d_{1j} \overline{X}_{ji} .$$

Again, this exactly mirrors the average derivate of the outcome with respect to $X_{1}$ when no confounding is present, as established by Eq. (10).

The same procedure (adding the mean of the covariate to the residual) can also be applied if covariates have been centered before the analysis but one would like to evaluate slopes with respect to the uncentered variable. Here, the derivative $\frac{{\overline{dY}_{i} }}{{du_{i} }}$ with respect to $u_{i}$ in (15) corresponds to the derivative $\frac{{\overline{dY}_{i} }}{{dX_{1i} }}$ in (10) with respect to (the centered) $X_{1}$. Considering $Y$ as a function of the uncentered rather than the centered version of $X_{1}$ simply means shifting the $Y$ curve to the right by the average $\overline{X}_{1}$, which is exactly the same shift that occurs when considering $Y$ as a function of $\tilde{u}$ rather than $u$. Algebraically, this can also be verified by the chain rule.

Finally, while our main interest lies in the slope of the relationship between the outcome and the first covariate, we may also want to interpret the intercept. The average intercept would be determined by setting $u$ (as well as product terms involving it) equal to zero and letting other variables equal their averages. Assuming no nonlinearities (i.e., that the *c* and *d* parameters are zero) and that covariates are zero on average, the average intercept in Eq. (13) becomes:

19 $$\overline{Y}_{i} \left( 0 \right) = a + b_{1} \left( {g + \mathop \sum \limits_{j = 2}^{J} h_{j} \overline{X}_{ji} } \right) + \mathop \sum \limits_{j = 2}^{J} b_{j} \overline{X}_{ji} = a .$$

This is obviously the same intercept as in Eq. (9), which describes the relationship between the outcome and the covariates.

In the event of nonlinearities, the average intercept in (13) becomes more complicated as squares and interaction terms between covariates appear as well. The average intercept in (13) will not quite correspond to an average intercept in Eq. (9), making interpretation more difficult. To give the average intercept a clearer interpretation, one possibility is to shift the entire fitted curve vertically so that the implied intercept corresponds to the estimated outcome for an individual in the reference group with $X_{1} = 0$, while keeping the slope of the curve unchanged. We employ this strategy in our empirical example, where there is some evidence of non-linear effects.

## Appendix 3

See Tables 4 and 5.

**Table 4:** Simulation results as in Table 1 but with the correlation between *X*_1_ and *X*_2_ increased to 0.9

Model	Imputation	Interaction
Average $\widehat{\Delta \alpha }$	3.001	3.001
Average $\widehat{\Delta \beta }_{1}$	1.003	1.003
Average $\widehat{\Delta \beta }_{2}$	0.493	0.493
Average $\widehat{\Delta \beta }_{3}$	− 0.000	− 0.000
Average standard error of $\widehat{\Delta \alpha }$	0.182	0.182
Average standard error of $\widehat{\Delta \beta }_{1}$	0.232	0.232
Average standard error of $\widehat{\Delta \beta }_{2}$	0.232	0.232
Average standard error of $\widehat{\Delta \beta }_{3}$	0.122	0.122
95% CI coverage $\widehat{\Delta \alpha }$	0.945	0.949
95% CI coverage $\widehat{\Delta \beta }_{1}$	0.939	0.947
95% CI coverage $\widehat{\Delta \beta }_{2}$	0.952	0.954
95% CI coverage $\widehat{\Delta \beta }_{3}$	0.944	0.948

Result in the first column were obtained from the imputation/prediction model outlined in steps 1–4 using bootstrap for statistical inference, whereas results in the second column were obtained from a standard regression model with interactions

**Table 5:** Simulation results as in Table 1 but with the correlation between *X*_1_ and *X*_2_ increased to 0.9 and *X*_2_ omitted from the heterogeneity analysis

Model	Imputation	Interaction
Average $\widehat{\Delta \alpha }$	3.013	2.947
Average $\widehat{\Delta \beta }_{1}$	1.438	1.400
Average $\widehat{\Delta \beta }_{3}$	0.033	0.012
Average standard error of $\widehat{\Delta \alpha }$	0.183	0.181
Average standard error of $\widehat{\Delta \beta }_{1}$	0.143	0.143
Average standard error of $\widehat{\Delta \beta }_{3}$	0.123	0.123

Results in the first column were obtained from the imputation/prediction model outlined in steps 1–4 using bootstrap for statistical inference, whereas results in the second column were obtained from a standard regression model with interactions. In the imputation/prediction model, *X*_2_ was omitted from the last step and in the regression model with interactions, no interaction between treatment and *X*_2_ was included

## Appendix 4

See Tables 6.

**Table 6:** Relative impacts of individual characteristics on the estimated effects of having BMI ≥ 30 versus BMI < 30 on HDL (ln mmol/l), according to a regression model with interactions

	Univariate (95% CI)	Multivariate (95% CI)
*Interaction effects*		
Risky alcohol consumption	0.911 (0.737–1.125)	0.906 (0.730–1.124)
Smoker	0.897 (0.825–0.975)	0.907 (0.832–0.988)
Physical activity score (10,000 units, centered)	1.013 (0.955–1.073)	1.009 (0.952–1.070)
Energy intake (1000 kcal/day, centered)	0.978 (0.935–1.023)	0.964 (0.915–1.015)
Primary education	1.000 (ref)	1.000 (ref)
Secondary education	1.011 (0.888–1.151)	1.018 (0.893–1.161)
Tertiary education	1.090 (1.009–1.177)	1.085 (1.003–1.174)
Male	1.025 (0.963–1.091)	1.057 (0.983–1.135)
Age 45–49	0.960 (0.881–1.046)	0.967 (0.884–1.058)
Age 50–59	1.000 (ref)	1.000 (ref)
Age 60–68	1.012 (0.952–1.076)	0.999 (0.936–1.065)
Main effect of obesity	–	0.857 (0.810–0.906)

Heterogeneous effects of obesity on HDL levels (ln mmol/l), based on interaction models where all variables previously used in “step 1–3” were entered as main effects and either one or all variables previously used in “step 4” were interacted with obesity. Since univariate models (including only one interaction term) were run separately, there is no common main effect of obesity to be reported. Effects are multiplicative, as the outcome is measured in logarithms and we have exponentiated coefficient estimates
